# Therapeutic resources used by physiotherapists for the relief of labor pain: a cross-sectional study

**DOI:** 10.61622/rbgo/2024rbgo99

**Published:** 2025-01-23

**Authors:** Alessandra de Campos Gonçalves, Giovana Garçoni Poli, Clara Maria de Araujo Silva, Ana Carolina Sartorato Beleza, Richard Eloin Liebano

**Affiliations:** 1 Universidade Federal de São Carlos São Carlos SP Brazil Universidade Federal de São Carlos, São Carlos, SP, Brazil.; 2 Department of Rehabilitation Sciences University of Hartford West Hartford CT USA Department of Rehabilitation Sciences, University of Hartford, West Hartford, CT, USA.

**Keywords:** Physical therapists, Pregnancy, Parturiation, Childbirth, Labor pain, Labor, obstetric, Pain management, Non-pharmacological resources, Surveys and questionnaires

## Abstract

**Objective:**

The aim of the study was to identify non-pharmacological therapeutic resources used by physiotherapists for pain relief during labor and childbirth.

**Methods:**

This is a cross-sectional study conducted from January to March 2021, followed the STROBE guidelines. It included Brazilian physiotherapists with a minimum of two years in obstetric care experience. Data were collected using a 33-item online questionnaire, which covered sociodemographic details and the utilization of non-pharmacological resources. Descriptive analysis was used to determine participant characteristics. Associations between sociodemographic variables, specialist titles, participation in scientific events, and methods for pain relief methods during childbirth were assessed using chi-square or Fisher’s exact tests. Data were analyzed using SPSS version 23.0, with a significance level set at 5% (p < 0.05).

**Results:**

A total of 114 Brazilian physiotherapists participated in this study. Participants chose to utilize non-pharmacological therapies and resources that are within the scope of physiotherapists’ practice for labor pain. Kinesiotherapy with the use of devices was the most employed technique for pain relief during the birthing process.

**Conclusion:**

The study highlights the prevalent use of non-pharmacological therapeutic resources, particularly kinesiotherapy with devices, among Brazilian physiotherapists for labor pain relief.

## Introduction

The process of parturition is a comprehensive definition that includes events from the onset of labor to birth, maximizing the inherent strength and physiology of both the woman and the fetus, and refraining from interventions unless the well-being or safety of both is at risk.^(1)^There is recognition that labor pain needs to be relieved, as it can exert a negative impact on both the mother and fetus.^(2)^Studies also point out that women often turn to cesarean surgery due to fear or previous negative experiences related to pain during labor.^(3)^Thus, when labor begins and the contractions are regular, pain can and should be relieved.

Pharmacological and non-pharmacological resources are available to relieve pain during the birthing process. Pharmacological resources are considered effective,^(4)^but can have side effects, such as maternal hypotension, a reduction in uteroplacental perfusion, fetal bradycardia, fever, pruritus, an increase in the requirement of oxytocin, prolongation of the second stage of labor, a greater rate of surgical birth and higher costs.^(5,6)^Non-pharmacological resources are an alternative that strengthen the anatomy of birthing women, enabling their active participation during labor and birth and posing few contraindications, risks and side effects compared to pharmacological resources.^(2,7)^

The main non-pharmacological resources described in the literature and used for the relief of labor pain are acupuncture/acupressure,^(8)^aromatherapy,^(9)^warm-water shower,^(10,11)^ warm-water immersion,^(12)^kinesiotherapy with the aid of devices,^(13,14)^transcutaneous electrical nerve stimulation (TENS),^(15)^mobility exercises and changes of position,^(16)^hypnosis,^(17)^ music therapy,^(18)^rebozo,^(19)^reflexology,^(18)^breathing and relaxation techniques^(18)^ and manual therapy.^(18)^However, the use of non-pharmacological methods for pain relief during labor in Brazil is not yet a widespread reality. The ‘Nascer no Brasil’ study indicates that 20% of women treated in private healthcare facilities and 29.2% of those treated in public healthcare services had access to these methods.^(20)^

To the best of our knowledge, no previous studies have investigated the therapies and non-pharmacological resources most used by physiotherapists to relief the pain of labor and childbirth. Considering the inclusion of physiotherapists at maternities, it is important to know which interventions are used, as such information can lead to several investigations, as well as to analyze whether such conducts are in accordance with the evidence available in the literature.

## Methods

A descriptive cross-sectional online study was conducted following the Strengthening the Reporting of Observational Studies in Epidemiology (STROBE). The data collection period took place from January to March 2021.

Brazilian physiotherapists with at least two years of experience with obstetric care were included in the study. Public dissemination resources (researcher profiles and laboratory pages on social media were used to invite physiotherapists to participate in the study. Invitations were also sent through ABRAFISM (Brazillian Association of Physiotherapy in Women’s Health) by email.

The questionnaire used in the present study was based on a bibliographic survey of recent clinical trials and literature reviews that addressed the use of non-pharmacological interventions to relieve the pain of labor and childbirth in the MEDLINE/PubMed, Cochrane, LILACS and Scopus databases. This survey was performed between August and December 2020.

The following therapies and resources were considered: acupuncture/acupressure,^(8)^aromatherapy,^(9)^warm-water shower,^(10,11)^warm-water immersion,^(12)^kinesiotherapy with the aid of devices,^(13,14)^ transcutaneous electrical nerve stimulation (TENS),^(15)^mobility exercises and changes of position,^(16)^hypnosis,^(17)^music therapy,^(18)^rebozo,^(19)^ reflexology,^(18)^breathing and relaxation techniques^(18)^and manual therapy.^(18)^Questions also addressed sociodemographic characteristics and the educational background of the participants.

To ensure the understanding of the questionnaire, a pretest step was performed with five physiotherapists who work in the field of obstetric physiotherapy and have the title of specialist in physiotherapy in women’s health. These individuals received a structured form available online, on which they were instructed to indicate suggestions and changes necessary for the adequacy of the data collection questionnaire. The physiotherapists were instructed to read the questionnaire first and subsequently complete the assessment form.

Data collection was performed using the questionnaire available online composed of 33 items and subdivided into two parts: 1) sociodemographic characteristics and educational background (20 items); 2) non-pharmacological resources employed (13 items).

A section of specific items was offered for the resources addressed on the questionnaire that had application protocols (TENS, manual therapy and kinesiotherapy with the aid of devices). After stating the use of these protocols, the participants were redirected to items that addressed the methods, criteria for indication or interruption, orientations and other forms of applicability of the resources.

Descriptive analysis was performed with the calculation of absolute and relative frequencies for the characterization of the participants. Associations between sociodemographic variables (title of specialist and regular participation in scientific events) and the resources used to relieve pain during the birthing process were determined using either the chi-square test or Fisher’s exact test. Data analysis was performed using SPSS version 23.0 (SPSS Inc., Chicago, IL, USA), with the level of significance set at 5% (p < 0.05).

This study was approved by the Human Research Ethics Committee of the Federal University of São Carlos 4.438.901/ certificate number (*Certificado de Apresentação de Apreciação Ética*): 37602920.9.0000.5504.

## Results

One hundred and thirty-four therapists answered the online questionnaire, 20 of whom were excluded for not having the minimum time of experience in obstetric physiotherapy (two years) or registration with a regional council. Thus, the sample was composed of 114 physiotherapists who actively worked in the field of obstetrics. Seventy-three professionals (64%) did not have the title of specialist in the field of Physical Therapy in Women’s Health as the highest educational degree. Ninety-one participants (79.8%) worked predominantly in clinical practice. Private clinics were the most frequent working location – cited by 75 respondents (57%). Besides obstetrics, 28 participants (24.6%) also worked with urology and gynecology. Table 1 displays the sociodemographic characteristics of the participants.

**Table 1:** Sociodemographic characteristics of physiotherapists who participated in study

Characteristics	n(%)
Sex	
Female	111(97.4) 3(2.6)
Male	
Age group	
20-29 years	41(36)
30-39 years	51(44)
40-49 years	17(14.9)
50 years or older	5(4.4)
Working time in the field of obstetric physical therapy	
1-3 years	47(41.2)
4-7 years	29(25.4)
8-11 years	14(12.3)
12-15 years	12(10.5)
Over 15 years	12(10.5)
Title of specialist	
Yes	41(36)
No	73(64)
Regular participation in scientific events (more than once per year)	
Yes	101(88.6)
No	13(11.4)

Table 2 presents the frequency of non-pharmacological resources used by the physiotherapists to relieve the pain of labor and childbirth. Kinesiotherapy with the aid of devices was reported by all participants in the study. Devices cited were: 99.1% (113) Swiss ball, birth stool and step 69.3% (79) wall bar, 50.00% (57) birth chair, 22.8% (26) rocking chair, 3.5% (4) bean ball, 0.09% (1) birth stool, step, rebozo and peanut ball. Non-pharmacological resources in the form of electrophysical agents also had considerable rates of use among the therapies, with warm-water shower, warm-water immersion and TENS reported by more than half of the participants (52.6%).

**Table 2:** Therapies and non-pharmacological resources used by Brazilian physiotherapists to relieve pain of labor and childbirth

Therapies and resources	Rate of use n(%)
Kinesiotherapy with the aid of devices	114(100)
Postural orientation	113(99.1)
Breathing and relaxation techniques	112(98.2)
Manual therapy	111(97.4)
Music therapy	85(74.6)
Warm-water immersion	77(67.5)
Rebozo	73(64)
Warm-water shower	72(63.2)
Aromatherapy	66(57.9)
TENS	60(52.6)
Acupuncture/Acupressure	43(37.7)
Reflexology	25(21.9)
Hypnosis	4(3.5)

TENS - transcutaneous electrical nerve stimulation

Table 3 displays the frequency of non-pharmacological resources that have application and/or use protocols, as well as the main times at which these methods are used and interrupted. Among the resources addressed on the questionnaire, kinesiotherapy with the aid of devices, postural orientations (changes of position), breathing and relaxation techniques, manual therapy, TENS and acupuncture/acupressure had a section of specific items so that professionals could report addressing the methods, criteria for indication and interruption, orientations and other forms of applicability for such therapies and resources.

**Table 3:** Report from professionals regarding application protocols and/or use of therapies and non-pharmacological resources

Therapies and resources	Time at which method is used n(%)	Interruption n(%)
Kinesiotherapy with aid of devices	Change in stage of labor, between contractions, according to position of fetus in pelvis, upon request by patient and pain/discomfort intensity 30(26.3)	Upon request by patient, transition to other resource or change in stage of labor 24(21.1)
Postural orientation	Between contractions, at changes in stages of labor, according to position of fetus in pelvis, upon request and pain/discomfort intensity 25(21.9)	Upon request by patient 29(25.4)
Breathing and relaxation techniques	At all times 43(37.7)	Upon request by patient 24(21.1)
Manual therapy	Upon request by patient, during and between contractions 34(29.8)	Upon request by patient 29(25.4)
TENS	Regardless of stage of labor and varying in accordance with professional assessment 13(11.4)	Upon request by patient and transition to other type of resource 11(9.6)
Acupuncture/acupressure	Upon request by patient, during and between contractions 10(8.8)	Upon request by patient 9(7.9)

Considering professional training and the use of non-pharmacological resources, associations were verified between holding the title of specialist and regular participation in scientific events with the choice of resources used. In these analyses, it was observed that individuals who obtained the title of specialist in women’s health recommended aromatherapy less than therapists without this title (p = 0.008). No significant associations (p < 0.05) were found between holding the title of specialist and other non-pharmacological resources. Similarly, the association between regular participation in scientific events and the choice of non-pharmacological resources was also not significant.

## Discussion

The aim of the present study was to identify non-pharmacological resources used by Brazilian physiotherapists to relieve pain during labor and childbirth. This study also enabled the determination of how physiotherapists in the field of obstetrics work during labor and childbirth, demonstrating their important inclusion on the multidisciplinary team at maternities.

Among the therapies and resources addressed on the questionnaire, kinesiotherapy with the aid of devices was the only therapy used by all participants, who declared the benefits of this method for relieving pain during the birthing process. The devices described by the professionals were Swiss ball, wall bars, birth stool, rocking chair, step, bean ball, among others. The birth ball stands out as one of the most widely used device, which agrees with data described in previous studies.^(13,14)^

A birth ball is commonly used by healthcare providers to relieve pain during labor and diverse mechanisms may explain how this relief occurs.^(13)^The gait control theory is currently one of the most widely cited mechanisms,^(13)^by which the activation of large-diameter afferent fibers reduces the activity of nociceptive fibers in the dorsal horn and spinal cord.^(21)^ This physiological response may occur through the movement of the pelvis over the ball during labor. The sitting position also reduces pressure on nerve fibers in the sacroiliac joint, alleviating pain in the lumbar region.^(13,22)^

Exercises based on the mobility orientation and changes of position were also frequently cited by the physiotherapists, chosen as an option for relieving pain by 99.1% of the participants. This result is compatible with findings described in a systematic review of the Cochrane Library, in which walking and changing positions during the first stage of labor presented important results with regards to the reduction in the duration of the birthing process, and the need for epidural anesthesia,^(16)^which indicated a possible analgesic effect without being associated with an increase in interventions or risk to the mother and infant. This modality uses gravity to assist in the descent of the fetus to the pelvis, thereby intensifying uterine contractions and assisting in the dilation of the cervix and the successful conclusion of the first stage of labor,^(23)^which can make the entire birthing process occur more quickly.

A warm-water shower can offer pain relief during the birthing process through the release of catecholamines (adrenaline and noradrenaline) and an increase in endorphins,^(1)^with a consequent reduction in anxiety and increase in satisfaction on the part of the mother.^(11)^Seventy-two respondents (63.2%) reported using warm-water showers as a resource for relieving the pain of labor. Despite the small number of studies on the resource, the warm-water shower achieves positive results in terms of the relief of labor pain.^(10,11)^The studies cited also reported significant reductions in the use of analgesics, offering strong evidence of the effects of this modality on relieving labor pain.^(10,11,24)^

Despite being considered of little accessibility in Brazil,^(2)^a warm-water bath as a non-pharmacological resource for the relief of pain during labor and childbirth has become increasingly common. Seventy-seven therapists (67.5%) reported using this resource in their clinical practice.^(25,26)^A systematic review conducted by Cluett and collaborators found that labor in water can reduce the need for epidural anesthesia, especially when immersion occurs in the first stage of labor,^(12)^ which can exert a positive analgesic effect during this period.

TENS is a non-pharmacological resource that has been used during the birthing process since the 1970s.^(27)^More than half of the respondents (52.6%) reported using TENS to relieve labor pain, although considerable divergence was found regarding the time at which the resource is employed and interrupted. Such divergence may be explained by the large number of application protocols. In a systematic review, Thuvarakan et al.^(15)^ concluded that TENS has significant effectiveness in the relief of labor pain, although the evidence is considered limited and of insufficient quality to judge the clinical effects. The authors stated that the risk of bias in the clinical trials analyzed was considered very high, indicating the need for further studies with adequate randomization methods and clearly stated blinding procedures.^(15)^

Forty-three respondents (37.7%) reported using acupuncture/acupressure as pain relief therapy, which is commonly combined with manual stimulation at different points of the body. A recent systematic review by Smith and collaborators pointed out that the quality of evidence for acupuncture/acupressure regarding the outcomes of studies addressing labor pain is low and clinical trials with better methodological quality are needed to investigate the effects of this resource on the relief of labor pain.^(8)^Acupuncture/acupressure techniques require training courses for use in Brazil. Thus, few general physiotherapists have knowledge regarding the application of such techniques, especially during labor and childbirth.

Despite not having established effectiveness,^(28)^76 respondents (57.9%) reported using aromatherapy as a non-pharmacological resource to relieve labor pain. However, specialists in physiotherapy in women’s health who acquired their title through (COFFITO [National Council of Physical Therapy and Occupational Therapy]/ABRAFISM [Brazilian Association of Physical Therapy in Women’s Health] recommended aromatherapy less than therapists without this title. Aromatherapy is outside the scope of the practice of specialists and is generally used by other healthcare providers who provide assistance to birthing women, such as midwives and doulas, which may explain this association. A systematic review conducted by Liao and collaborators found that aromatherapy significantly relieved pain in nulliparous women during the first stage of labor.^(9)^The authors also pointed out that the fact that this resource only presented significant results in nulliparous women constitutes a major limitation of the study.^(9)^

The use of relaxation techniques involving breathing or music can contribute to a reduction in pain intensity.^(18)^These two resources were used by 98.2% and 74.6% of the respondents, respectively. Breathing techniques and music therapy can assist birthing women feel in control and satisfied with childbirth, but the considerable variation in the types of techniques used hinders a conclusion regarding whether these methods indeed assist in relieving labor pain, which lowers the quality of the evidence.^(18)^

Rebozo is a non-pharmacological resource involving the use of a long cotton fabric and was used by 73 (64%) of the 114 respondents. Although explored little in the scientific literature and requiring further studies for a better understanding of its possible effects during labor,^(19)^rebozo is considered a safe, low-cost resource that does not pose any major risk to the mother or fetus,^(19,29)^which may explain its use by more than half of the participants.

Massage performed in the first stage of labor can reduce pain intensity during the entire birthing process.^(5)^ Manual therapy was reported by 111 of the respondents (97.4%), whereas reflexology was reported by only 25 (21.9%). Massage is generally performed in the lumbosacral region of birthing women^(18,24)^and can be executed using classic techniques. The use of massage by the therapists who answered the questionnaire is in agreement with findings described in a systematic review conducted by Smith and collaborators, even though part of the evidence is considered limited with regards to the reduction in pain during the birthing process.^(18)^Moreover, evidence on the effects of reflexology regarding pain relief is insufficient.^(18)^

Besides characteristics related to the use of therapies and non-pharmacological resources during labor and childbirth reported by the participants, this study also makes important contributions to knowledge on how these physiotherapists provide care at maternities. No studies were found in the literature on the assessment or other characteristics of the inclusion of physiotherapists to the multidisciplinary team at maternities in Brazil and there is no consensus on the quantity of therapists who work in the field of obstetrics. This study also raises issues regarding what therapies and/or non-pharmacological resources can or cannot be considered physiotherapeutic conduct or simply obstetric assistance techniques.

Considering the presented results, it is important to emphasize one of the strengths of this study, which is being the first survey to investigate the use of resources by physiotherapists in Brazil. Thus, it highlights the importance of integrating physiotherapists into the team that assists the parturient, to facilitate labor progress, alleviate pain, and promote a positive birthing experience.^(30)^Finally, we acknowledge as limitations of this study its online nature, as participants may have found the data collection form overly lengthy, and its self-administered format, which precluded the clarification of any doubts. Another limitation is that while all participants had at least two years of experience in obstetrics, the extent of this experience in terms of the number of patients they cared for was not controlled, and physiotherapists were asked to consider their clinical experience in years.

## Conclusion

Most of the Brazilian physiotherapists who participated in the present survey opt for the use of non-pharmacological resources to alleviate pain during labor and childbirth. The results of this study emphasize the prevalence, particularly kinesiotherapy with devices, among Brazilian physiotherapists.
